# Improvement of stem cell performance by supplementation with metabolic enhancers

**DOI:** 10.1186/1753-6561-5-S8-P13

**Published:** 2011-11-22

**Authors:** Abi M Abitorabi, Christopher Wilcox

**Affiliations:** 1 GIRUS Life Sciences, Inc., Sunnyvale, CA, 94085, USA; 2 Sheffield BioScience, Beloit, WI, 53511, USA

## 

**Figure 1:**
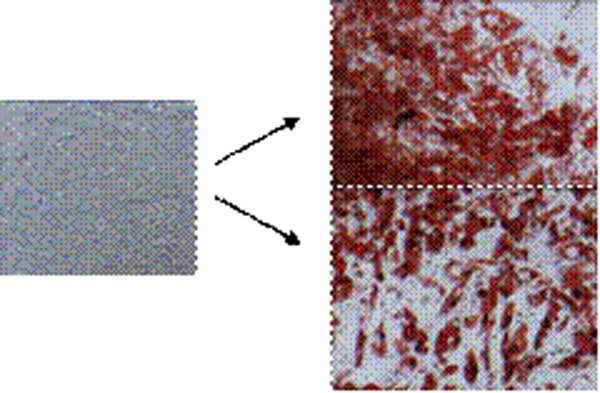
Human MSC expanded in GEM Novo containing RS Novo can later differentiate into adipocytes as demonstrated here by Oil Red O staining.

Practical culture methods for expansion of human stem cells are needed for research and industrial applications. In general, ingredients of known and unknown composition have been used in stem cell cultures based on past studies and practices. To harness the substantial potential of stem cells in treating human diseases, products with improved characteristics are needed to support the manufacturing of stem cells for cell therapy use. Safety, definition, cost and consistency are key considerations for all new stem cell products. Based on data mining and cell-based screens, we have designed the RS Novo™ defined small molecule metabolic enhancers for expansion of human embryonic (ESC), mesenchymal (MSC) and hematopoietic stem cells with limited differentiation. Cell growth, viability, phenotype and stem cell potential were endpoints of interest in our studies. Our aim was to minimize media components that would “trigger” stem cells into differentiation, apoptosis, and necrosis. We also aimed to minimize undefined components like serum that could introduce inconsistencies. Here, we cover some RS Novo™ results with human MSC and ESC and introduce the GEM Novo™ serum free medium for a more complete culture system for stem cell expansion. Cells grown in this culture system maintained their stem cell phenotype and potencies. For example, at different passages, human ESC H7 clone maintained the Oct-4 marker of undifferentiated cells more consistently in this system versus a culture system optimized by others (Table 1), and human MSC expanded in GEM Novo containing RS Novo and then transferred to differentiation media could differentiate into adipocytes (Figure 1). Altogether, this new culture system provides a consistent, high performance condition for human stem cell expansion.

**Table 1:** Consistent marker expression at different passages of human ESC culture in GEM Novo medium containing RS Novo, determined by mean fluorescence intensity (MFI) of Oct-4 staining by flow cytometry.

	Marker	Control Optimized Conditions MFI	GEM Novo & RS Novo MFI
Passage 3	Oct-4	75.8	81.5

Passage 8	Oct-4	66.1	81

